# Epigenetic mechanisms in stem cell therapies for achilles tendinopathy

**DOI:** 10.3389/fcell.2025.1516250

**Published:** 2025-03-13

**Authors:** Zheyang Yuan, Zheyu Yao, Xufeng Mao, Xiang Gao, Sengyi Wu, Haijiao Mao

**Affiliations:** Department of Orthopaedic Surgery, The First Affiliated Hospital of Ningbo University, Ningbo, Zhejiang, China

**Keywords:** Achilles tendinopathy, epigenetics, stem cell therapy, DNA methylation, histone modification, non-coding RNAs

## Abstract

Achilles tendinopathy (AT) is a chronic degenerative tendinopathy that affects people’s daily lives. Multiple clinical studies have found that current conservative treatments fail to promote quality tendon healing. Recent studies have found that stem cell therapy can target pathophysiological changes in the tendon by replenishing tendon-derived cells, promoting extracellular matrix (ECM) remodeling, and modulating the inflammatory response to improve the microenvironment of Achilles tendon regeneration. And epigenetic modifications play an important role in stem cell fate determination and function. In this review, we provided a brief overview of the biological properties of relevant stem cells. The influence of epigenetic modifications on stem cell proliferation, differentiation, and immune regulatory function in the treatment of AT was also explored. We focused on gene regulatory mechanisms controlled by DNA methylation, histones and non-coding RNAs including microRNAs, circRNAs and long non-coding RNAs. We also discuss the current challenges faced by stem cell therapies in treating AT and their potential solutions. Further research in this area will provide a more comprehensive epigenetic explanation for stem cell therapy for AT, leading to the development of stable, safe and effective stem cell therapies.

## 1 Introduction

Achilles tendinopathy (AT) is very common among athletes and people engaged in repetitive mechanical activities, severely affecting people’s daily activities. The incidence of AT is about 2-3 per 1,000 adults, while in the active sports population the cumulative lifetime prevalence rate can even increase to more than 50% (Van Der Vlist et al., 2021). AT is a non-fracture chronic degenerative tendon disease. Clinical symptoms include pain, swelling and limitation of movement of the Achilles tendon and adjacent tissues (Steinmann et al., 2020). Microscopically attributable to damage to the tendon matrix and the accumulation of secreted substances such as cytokines, chemokines, and inflammatory mediators (Millar et al., 2021). Injured tendons have a limited ability to repair due to their low cell density, and poor innervated blood vessels (Tsutsumi et al., 2022). The main goal of treatment is to relieve pain, promote tendon repair and restore Achilles function (Millar et al., 2021). Eccentric strengthening should be recommended first, followed by topical or oral anti-inflammatory medications, local injections such as platelet-rich plasma (PRP), shock-wave therapy and orthosis,etc (Cooper, 2023). Surgical intervention is recommended for severe cases of AT that are unable to ambulate or if there is no relief of pain and motor function after at least 3 months of conservative treatment (Cooper, 2023). However, while conservative treatment appear to alleviate the symptoms of pain in the short term (up to 3 months), with unsatisfactory long-term results (beyond 12 months) (Wilson et al., 2018; Malmgaard-Clausen et al., 2021; Rabusin et al., 2021; Van Der Vlist et al., 2021; Cooper, 2023). Existing treatments have limited impact on AT pathological repair and may not achieve high quality healing of the tendon, because they lack the ability to promote intrinsic tendon regeneration and effectively remodel the injured tendon tissue (Li et al., 2024; Wei and Lu, 2021). This not only reduces the patient’s quality of life such as: participation in sports, recreation and work, but is also associated with increased psychological stress, depression and anxiety.

**FIGURE 1:**
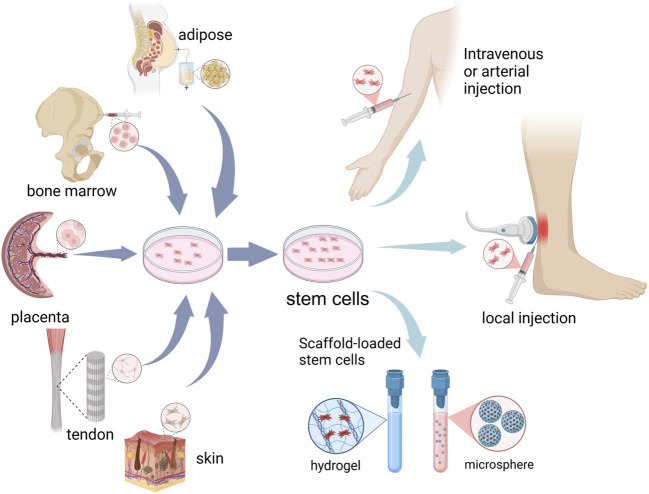
Schematic representation of cell sources and administration routes for stem cell therapy in Achilles tendinopathy.

Stem cells have gained attention by showing excellent therapeutic potential for degenerative and inflammatory diseases. Their minimal immunogenicity opens the door to applications in both autologous and allogeneic contexts (Walewska et al., 2023). Stem cell therapy refers to the provision of cell populations that have the capacity to self-renew and differentiate into multiple cell lineages to achieve tissue homeostasis and regeneration through stimulation, modulation, and regulation of the endogenous stem cell population and/or replenishment of the cellular pool of injured tissues (Si, 2019; Hoang et al., 2022). Recent advancements in stem cell technology have ushered in a new era for the treatment of AT, showing excellent prospects. Stem cell can replenish tenocytes, promote extracellular matrix remodeling, modulate the inflammatory response, etc., thus improving the microenvironment of the Achilles tendon and promoting repair (Figure 1).

## 2 Stem cell therapy

### 2.1 Source and biological characteristics of stem cells

Stem cells undergo upregulation of tendon-related genes and tendon extracellular matrix (ECM) markers as well as massive deposition of ECM, inducing differentiation into tendon stem cells and tenogenic differentiation (Costa-Almeida et al., 2019; Donderwinkel et al., 2022). Currently, the main types of stem cells for AT (Table 1) include: (a) Mesenchymal stem cells (MSCs), the most widely used sources are adipose MSCs. Bone marrow and placental MSCs have been employed in phase I/II clinical studies of human AT (Ilic and Atkinson, 2014; Goldberg et al., 2018; 2024; Thueakthong et al., 2021). It holds the greatest promise for clinical application due to its easy accessibility, diverse sources, minimal harm to the human body, and its capacity to widely expressed tendon-related genes. (b) Tendon-derived stem cells (TDSCs), extracted from Achilles tendon, is widely applied in AT in animals. However, the difficulty of obtaining human tendon tissue and low cell numbers limit its extraction and application in the clinic. (c) Induced pluripotent stem cells (iPSCs), iPSCs have emerged as a promising cell source for tenogenic differentiation, owing to their remarkable capacity for unlimited proliferation and their versatility in differentiating into various cell types (Yamanaka, 2024). iPSCs can be induced from adult human dermal fibroblasts and other human somatic cells, which means that one of their significant advantages is that they can be non-invasive to the body during the extraction process (Takahashi et al., 2007). However, due to its complex induction process (Liuyang et al., 2023), such as transcription and translation, a mature culture system for the differentiation of iPSCs towards Achilles tenocytes needs to be further evaluation in terms of clinical applications.

**TABLE 1:** Advantages and limitations of stem cell commonly used in AT.

Cell type	Origin of cells	Clinical readiness	Advantages	limitations	References
MSCs	Adipose	Less invasive	Rich source, low immunogenicity, more expression of tenogenic genes, more clinical studies conducted	Multidirectional differentiation capacity, ethical issues with placenta origin	Kern et al. (2006), Preynat-Seauve and Krause (2011), Watanabe et al. (2021)
	bone marrow	Highly invasive			
	placenta	Non-invasive			
TDSCs	Achilles tendon	Highly invasive	Low immunogenicity, most abundant expression of tenogenic genes	low source: few stem cells in Achilles tendon tissue, difficult to expand	Preynat-Seauve and Krause (2011), Costa-Almeida et al. (2019)
iPSCs	Skin(mature differentiated cells)	Less invasive	Rich source, low immunogenicity, expansion requires fewer primary cells, theoretically iPSCS can be amplified Characterized by juvenile	Complex differentiation steps, iPSCs need to be induced into iPSC-MSCs before transplantation	Winston et al. (2019), Nakajima et al. (2021), Yamanaka (2024)

#### 2.1.1 Mesenchymal stem cells (MSCs)

MSCs can be isolated from virtually every tissue in the body, with the most common source being adipose, followed by bone marrow, placenta, etc (Si, 2019). The extracted adipose tissue was finely minced and subsequently digested with Type I Collagenase at room temperature with permanent shaking for a few hours before isolating the primary MSC (Melzer et al., 2021). Primary bone marrow-derived MSCs were isolated using the whole bone marrow adherent method and non-adherent cells were subsequently removed after 24 h (Wei et al., 2023). MSCs highly express CD29, CD73, CD90, and CD105 but lack expression of the surface molecules CD45, CD34, CD14, CD11b, CD79a, CD19, and HLA-DR (Si, 2019; Wu, 2023; Goldberg et al., 2024). *In vivo*, the safety of autologous MSCs injections for treating AT has been well-documented; however, the underlying mechanisms of action remain not fully understood (Goldberg et al., 2024).

*In vitro*, MSCs are often used as comparison samples for characterisation of tendon stem cells and can express a diverse array of tenogenic differentiation markers, including scleraxis (SCX), tenomodulin (TNMD), collagen I, II, and III (Col I,II, and III), decolin (DCN) and biglycan (Bgn), but at lower levels than TDSCs (Costa-Almeida et al., 2019). The application of MSC therapy to treat AT is now widely used and validated. Preclinical study shows significant results of *in situ* implantation of autologous bone marrow mesenchymal stem cells for treatment of AT (Smith et al., 2013). In comparing various sources of MSCs, Myrto Bami et al. found that synovial-derived tenomodulin had the highest level of expression, had a high proliferative capacity, and showed superiority in tendon generation (Bami et al., 2020). In addition, MSCs are highly sensitive to their environment, including adaptation to the extracellular environment. In the Achilles tendinopathy microenvironment, this adaptation can increase cellular potency (Wang et al., 2020; Melzer et al., 2021).

#### 2.1.2 Tendon-derived stem cells (TDSCs)

In 2007, Bi et al. first identified and isolated TDSCs from human and mouse tendon tissues (Bi et al., 2007). TDSCs were derived from biopsies of the Achilles tendon tissue (Yin et al., 2020). The Achilles tendon was removed from the peritendinous tissue, cut up and digested with Type I Collagenase for several hours. Then, TDSCs were induced by resuspending the cell suspension obtained after filtration in a specialised medium for tendon stem cells (Mao et al., 2022). TDSCs stem cells have typical features of MSCs, such as typical surface antigens, self-renewal, clonality and the potential for differentiation into adipogenic, osteogenic, and chondrogenic lineages (Schneider et al., 2018; Guo et al., 2023). TDSCs expressed CD44, CD90, CD90.1, CD105, CD146, Stro-1, Oct-4, nucleostemin, and SSEA-1, but not CD31, CD45, CD34, CD18, CD106, CD117, CD144, and Flk-1 (Schneider et al., 2018). Our previous studies have cultured TDSC with uniform long spindle cell morphology (Mao et al., 2022; Mao et al., 2024; Zhang et al., 2024). We have verified that these cells expressed CD29 (100%), CD44 (98.8%) and CD90 (89.2%) and CD31 and CD34 were negative. In addition, tenogenic differentiation markers such as SCX, TNMD, COL Ⅰ and COL Ⅲ were upregulated during tenogenic differentiation (Mao et al., 2022). TDSCs are the ideal cell type for application in AT. It is homologous to tenocytes and expresses high levels of SCX, TNMD and tenascin-C (TNC) than MSCs during differentiation (Lu et al., 2023a). It has the potential to spontaneously differentiate into tendon cells including tendonocytes and ECM formation improve the cellular matrix, influence angiogenesis and collagen synthesis, as well as regulate the metabolism of the Achilles tendon microenvironment.

#### 2.1.3 Induced pluripotent stem cells (iPSCs)

iPSCs can be derived through the chemical reprogramming of human somatic cells, such as fibroblasts. The earliest and most classical iPSCs were generated by using four transcription factors (OCT4, SOX2, KLF4 and c-Myc) (Takahashi et al., 2007). Owing to the intricate processes involved, such as transcription and translation, controlling transcription factors once they are delivered into cells presents a significant challenge. In contrast, strategies in which non-genetic and exogenous stimuli such as small molecules induce iPSCs regulation have been welcomed. Small molecules, typically less than 900 Da in molecular weight, are capable of permeating cell membranes. These molecules can directly interact with intracellular and cell-surface proteins, which are crucial for cell signaling pathways and epigenetic modifications, including changes to chromatin, histones, and DNA (Wang J. et al., 2023; Liuyang et al., 2023 optimised the original protocol using VTP50469, AKT kinase inhibitor, CX4945, and 5-iodotubercidin to rapidly and efficiently induce the generation of iPSCs (Liuyang et al., 2023). In repairing Achilles tendon, iPSCs are usually induced into iPSC-MSCs first, and then cytokines and Achilles tendon-related markers are added to match the microenvironment for tenogenic differentiation. Can Zhang et al. induced the generation of iPSCs from human foreskin fibroblasts. Then by changing the medium composition (including DMEM-low glucose, Glutamine, NEAA, human recombinant bFGF, ascorbic acid and 10% FBS), hiPSCs were differentiated towards MSCs. Ultimately, hiPSC-MSCs were cultured on scaffolds mimicking the structure of tendon ECM for further tenogenic differentiation (Zhang C. et al., 2015a). In addition, it was demonstrated that human iPSCs can directed differentiation of tenocytes by modeling human somite development *in vitro* (Nakajima et al., 2018; Nakajima et al., 2021). This process is characterized by the general expression of SCX, Mohawk (MKX), COL Ⅰ and POSTN (Nakajima et al., 2021).

### 2.2 Stem cell delivery in the treatment of achilles tendinopathy

When considering the use of stem cell therapies, it is important to explore the best culture and delivery methods for stem cells. Due to the rich matrix and lack of vascularity of the Achilles tendon, it is difficult to accumulate a therapeutic dose of cells to repair Achilles tendon injuries by intravenous injection. Currently stem cells in the Achilles tendon are preferred to be injected *in situ* to maintain the concentration of stem cells in the localized area (Han et al., 2024). The stem cells used in treatment are isolated or induced from human tissue and expanded in standardized cultures up to a dose of 4–20*10^6^, which is considered to be the appropriate dose for efficacy (Goldberg et al., 2024). A suspension of purified stem cells was administered into a culture medium and subsequently injected along the length of the Achilles tendon through a percutaneous approach under local anesthesia. This procedure was performed by an experienced and trained physician using ultrasound guidance to ensure precision and safety. After determining the location of maximum anterior-posterior thickness of the tendon, the needle was inserted from proximaldistal to distal and withdrawn simultaneously during the injection (Goldberg et al., 2018; Goldberg et al., 2024). After implantation, patients needed to be observed for at least 2 h for any early adverse reactions. And follow-up phone calls were required.

In addition, many studies have inoculated stem cells onto scaffolds to promote stem cell survival *in vivo*, as well as to increase the potential for stem cell migration and differentiation into tendon cells. Achilles tendon ruptures require surgical treatment and can be treated with loaded stem cells such as surgical sutures and surgical patches to promote early healing of the Achilles tendon (Yao et al., 2012; Schon et al., 2014), whereas Achilles tendinopathy is typically treated with injections. Hydrogels are widely used as ideal artificial extracellular matrices for 3D cell cultures for the treatment of AT with loaded stem cells. Zilu Ge et al. found that DNA hydrogels provide a suitable artificial ECM microenvironment for the *in vivo* survival of TDSCs, and they can also restrict TDSCs in tendons and prolong their retention time, which can help to promote the repair of AT (Ge et al., 2023). The decellularised extracellular matrix of tendon and porcine small intestinal submucosa (SIS) has also been widely accepted as an ideal matrix for tendon regeneration, which provides an environment more conducive to the proliferation of TDSCs (Ning et al., 2021; Mao et al., 2022).

## 3 Key factors affecting the effectiveness of stem cell repair

Repair of AT injuries involves complex biological processes with multiple overlapping stages, including inflammation, proliferation and remodeling (Schneider et al., 2018; Millar et al., 2021; Korntner and Zeugolis, 2019). The process of tendon healing encompasses three overlapping phases: inflammation, proliferation, and remodeling. During the inflammatory phase, inflammatory cells secrete substantial amounts of cytokines which promote cell migration and the formation of new blood vessels. During proliferation stage, macrophages release growth factors to direct cell recruitment and activity, tendon fibroblasts and intrinsic tenocytes are recruited to the injury site to produce Col III, fibronectin and ECM components (e.g., proteoglycans) (Schneider et al., 2018). The second phase is the repair process, which is guided by tenocytes and macrophages in a number of regenerative activities including fibroblast proliferation, ECM oversynthesis and tenogenic differentiation. Finally, the newly produced Col Ⅰ recombines to form healing tissue (Zhang et al., 2021; Li et al., 2023). Here, we summarize some of the important factors in the regeneration process, including alterations in the extracellular matrix (ECM), inflammatory response, and accumulation of reactive oxygen species (ROS) in AT, which may affect the loss or depletion of native TDSCs, as well as the transplanted stem cells.

### 3.1 Changes in ECM

The basic component of tendon consists of Col Ⅰ (approximately 60%–85% of its dry weight), while the remaining components including proteoglycans, proteoglycans, glycosaminoglycans, glycoproteins and other collagen subtypes, such as collagen III, V and XII (Costa-Almeida et al., 2019; Millar et al., 2021). The stem-cell niche has been defined as a specialized microenvironment that shelters stem cells and sustains a delicate equilibrium between quiescence, self-renewal, and cell fate commitment. This three-dimensional architecture is intricately composed of various cells, cytokines, and the ECM (Bi et al., 2007). TDSCs are primarily found within the stem cell niches of the Achilles tendon. These niches are predominantly situated in the tendon fascicles, the peritendinous tissues, as well as the vascular regions of the tendon (Schulze-Tanzil et al., 2022). The niche is predominantly comprised of ECM. Alterations in the composition of the ECM can significantly impact the size of the TDSC pool (Bi et al., 2007). In response to external stimuli such as mechanical loads, inflammation or various biological factors, specific TDSCs are activated to initiate the process of self-renewal or differentiation (Guo et al., 2023). Biglycan (Bgn) and fibromodulin (Fmod) are considered essential components of this ecological niche. The absence of Bgn and Fmod leads to reduced expression of SCX and TNMD, thereby affecting the differentiation of TDSCs through the regulation of bone morphogenetic protein (BMP) signaling. This shift disrupts the balance between ligament formation and bone formation in TDSCs, ultimately leading to tendon heterotopic ossification (Bi et al., 2007). The expression of key tendon genes, such as SCX, diminishes over time in culture due to the lack of tendon ECM in vitro conditions. This deficiency can impair the self-renewal and differentiation abilities of TDSCs (Webb et al., 2016). Cultured with young decellularised extracellular matrix (DECM), ageing TDSCs with impaired ability to regain viability, proliferation and tenogenic differentiation, and reduced expression of senescence-associated markers in ageing TDSCs (Dai et al., 2019).

### 3.2 Inflammation

The important role of inflammation in the pathogenesis of AT is now well established. Various subtypes of immune cells and inflammatory mediators have been identified as key factors in both the development and progression of this condition (Millar et al., 2017). Following tissue damage, immune cells and activated tenocytes release elevated levels of pro-inflammatory cytokines, including tumor necrosis factor-alpha (TNF-α), IL-1β, IL-6 and interferon-gamma (IFN-γ). This cascade of inflammatory mediators disrupts both the microstructure and composition of the tendon, leading to significant functional impairment (Smith et al., 2023; Han et al., 2024). Inflammatory cytokines, such as IL-1β, compromise the microstructure and composition of the tendon ECM and diminish its biomechanical properties. This occurs through the induction of inflammatory mediators like cytosolic phospholipase A2 (cPLA2), cyclooxygenase-2 (COX-2), and prostaglandin E2 (PGE2), as well as by enhancing the expression or activity of matrix metalloproteinases (MMPs), including MMP1, MMP3, and MMP13, within tenocytes (Zhang K. et al., 2015b; Han et al., 2024). IL-1β alters the microenvironment of the TDSCs niche by suppressing the expression of Bgn and Fmod, consequently impacting stem cell proliferation and tenocytes differentiation (Zhang K. et al., 2015b). In the inflammatory microenvironment, both bone deposition and phosphatase levels in TDSCs are elevated, alongside the activation of the Smad and NF-κB, leading to osteogenic differentiation of TDSCs to form Achilles tendon heterotopic ossification (HO) (Huang et al., 2022). The inflammatory microenvironment also enhances osteogenic differentiation of MSCs (Dighe et al., 2013). Inflammation reduces TNC and SCX expression in TDSCs but promotes proliferation and migration of TDSCs, leading to fibrotic scarring of the Achilles tendon (Jiao et al., 2022; Dou et al., 2024).

### 3.3 Reactive oxygen species (ROS)

Many factors, such as overloading, repetitive mechanical stimulation, ischaemia-reperfusion injury, hyperthermia in exercising tendon, release of inflammatory cytokines, drug exposure (fluoroquinolones, steroids, anaesthetics), and metabolic factors (hyperglycaemia, hyperlipidaemia, and hypercholesterolaemia), can contribute to the production of endogenous ROS by the mitochondria of cells within the Achilles tendon tissue (Li et al., 2020; Li et al., 2023; Liu et al., 2020; Liu et al., 2021a.; Lui et al., 2022). Moderate levels of ROS promote cell proliferation and differentiation, whereas excessive ROS induce apoptosis or autophagy due to oxidative damage to lipids, proteins and DNA (Li et al., 2020). Mitochondrial dysfunction and ROS production impaired colony formation, stemness marker gene expression and multilineage differentiation capacity of TDSCs as well as showing inhibited the migration and proliferation of cells (Sun et al., 2020). In addition, ROS are directly cytotoxic to ECM components. ROS oxidise susceptible amino acids in collagen, altering protein conformation, and also activate MMPs, which in turn synergistically increase cytotoxicity (Bisaccia et al., 2019). Inhibiting the activation of the NF-κB pathway in TDSCs not only reverses the ROS-induced loss of stemness by sustaining the expression levels of SOX2, OCT4, and CD146 but also enhances the tenogenic differentiation potential of TDSCs through the promotion of SCX and TNMD expression (Lu et al., 2023b). Moreover, ROS mediate several critical signaling pathways, including the induction of apoptosis and autophagy in TDSCs via the activation of the AKT/FOXO1, as well as the activation of NF-κB signaling, which inhibits the tenogenic differentiation of TDSCs (Li et al., 2019; Li et al., 2020).

## 4 Epigenetic mechanisms regulating stem cell activity

**FIGURE 2:**
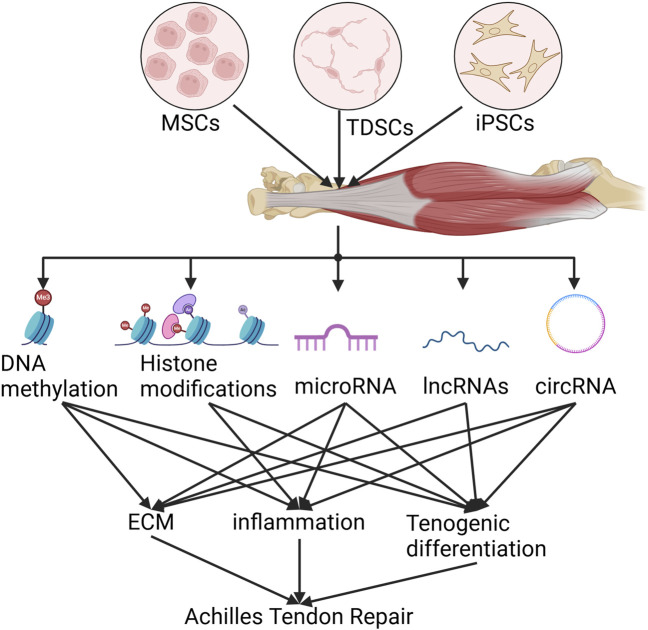
Schematic epigenetics of stem cell therapy for Achilles tendinopathy.

In 1968, C.H. Waddington coined the term “epigenetics”, defined as “the interactions and causal relationships between genes and the products of genes that determine phenotype” (Waddington, 1968). We now know that epigenetic mechanisms transmit the inheritance of gene expression patterns by adjusting chromatin (the physical form of our genetic information) without altering the underlying DNA sequence (Allis and Jenuwein, 2016). Epigenetic modifications have traditionally been thought to include chemical modification of cytosine bases in DNA and histone packaging proteins. The discovery of microRNAs in the late 1990s, followed by the detailed elucidation of the RNA interference mechanism, introduced a new dimension to this field (Riasat et al., 2020). In this section, we will specifically describe the epigenetic modifications that affect stem cell proliferation and differentiation in the treatment of AT (Figure 2). The focus will be on gene regulatory modifications governed by DNA methylation, histone modifications, RNA, and various non-coding RNAs.

### 4.1 Chromosome-associated epigenetic modifications

#### 4.1.1 DNA methylation

**FIGURE 3:**
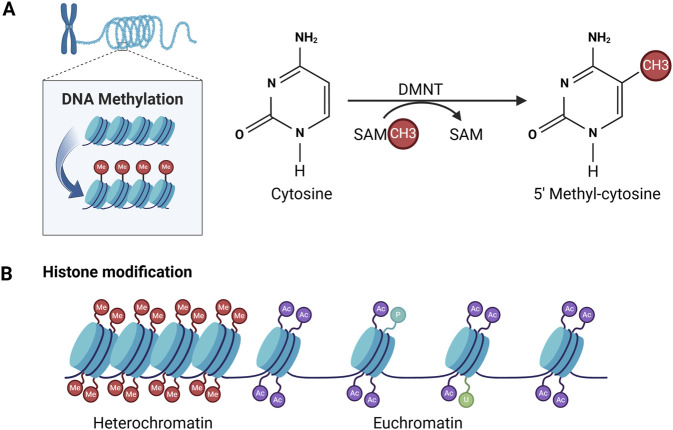
Patterns of DNA methylation and histone modifications mediating epigenetic alterations. **(A)** DNA methylation is achieved by adding a methyl group (-CH3) to cytosine. This process is catalysed by DNA methyltransferases (DNMTs). **(B)** Histone modifications include methylation (Me), acetylation (Ac), phosphorylation (P), ubiquitination (U).

DNA methylation at the fifth position of cytosine (5 mC), as a stable epigenetic mark, acts mainly as a transcriptional repressor (Kim and Costello, 2017; Thankam et al., 2019). DNA methylation is catalyzed by dNA methyl transferases (dNMTs), which removes methyl groups (-CH3) from cytosine residues on S-adenosyl methionine (SAM), particularly on CpG dinucleotide sequences, resulting in 5-methylcytosine residues (Figure 3) (Thankam et al., 2019). Approximately 70% of gene promoter sites are associated with CpG islands (CGIs) responsible for transcriptional initiation (Walewska et al., 2023).

Inflammatory responses can affect Achilles tendon healing by inducing epigenetic changes. Hypermethylation is the main methylation modification. cAMP Response Element-Binding Protein 1 (CREB1) upregulation induces TNF-α and IL-6-related pro-inflammatory responses. This involved 67 genes expressing hypomethylation/upregulation that were significantly enriched in multiple pathways, including gap junction formation, melanogenesis and PI3K-Akt signalling (Lee et al., 2022). Inflammatory cytokines inhibit the expression of genes and proteins, including SCX and TNMD, thereby impairing tenogenic differentiation of stem cells and affecting the quality of tendon healing (Dai et al., 2019). Five other genes (Leprel2, Foxf1, Mmp25, Igfbp6, Peg12) have also been identified as hypomethylated with strong correlation to multipotent mesenchymal cells, fibroblasts, tendon, and/or extracellular matrix (Trella et al., 2017). Among them, Leprel2, Foxf1, and Mmp25 exhibited functions closely related to collagen fibers. Igfbp6 is thought to play an important role in immunity and inflammation, including the regulation of proliferation, apoptosis, angiogenesis, cell migration and fibrosis progression (Liso et al., 2022). In addition, the PI3K/Akt signalling pathway is associated with maintaining the pluripotent state of stem cells and sustaining self-renewal capacity (Bechard et al., 2012). DNA methylation has also been demonstrated to inhibit the spontaneous differentiation of MSCs in culture, whereas removal of the methyl group resulted in a shift towards osteoblastic and adipogenic Differentiation (Webb et al., 2016).

#### 4.1.2 Histone modifications

Histones are the primary protein constituents of chromatin, playing a crucial role in condensing DNA into compact structures known as nucleosomes. Each nucleosome is formed by an octamer, comprising two molecules each of the four histone proteins (H2A, H2B, H3, and H4), which are wrapped with roughly 147 base pairs of DNA (Riasat et al., 2020). Amino acid residues at the N-terminus of histones are susceptible to post-transcriptional modification. To date, four mechanisms in histone modification have been best described and functionally characterised, namely, acetylation, methylation, ubiquitination and phosphorylation (Figure 3). Histone modifications are important for transcriptional regulation, and combinations of histone modifications in promoter regions can determine the epigenetic state of a cell, thereby promoting gene activation or repression (Thankam et al., 2019).

Over-activation of the NF-κB pathway leads to a low-grade inflammatory infiltrate, which can cause TDSCS senescence or aggravated cellular hypoplasia, whereas lowering the levels of H3K9me3 and H3K27me3 could reverse TDSCS aging by inhibiting the over-activation of NF-κB (Jin et al., 2023). Under normoxic conditions and/or in the absence of mechanical stimulation, amplification of TDSCs was accompanied by an overall decrease in histone methylation, a decrease in SCX nuclear binding, and spontaneous differentiation, with a gradual decrease in the stemness of TDSCs. Expression of “stemness” genes such as Oct4 and Sox2 is significantly reduced in successive generations of MSCs. Corresponding to these changes are epigenetic dysregulations, particularly histone H3 acetylation, which is closely associated with MSC senescence and spontaneous osteogenic differentiation (Li et al., 2011). Can Zhang et al. found that elevated levels of histone acetylation in the TGF-β signalling pathway (Tgfb1 and Tgfb2 genes) contribute to the maintenance of phenotypic properties of TDSCs (Zhang et al., 2018b). The expression of MKX, Eya1, HoxA11 and DCN was upregulated in TDSCs with elevated histone acetylation, whereas genes related to osteogenic (Runx2) and chondrogenic (Sox9) profiles were virtually unchanged (Zhang et al., 2018a).

In addition, H3K9MTase G9a may play an important role in regulating the differentiation and growth of TDSCs, and the proliferation of G9a-deficient TDSCs was significantly reduced. Furthermore, the expression levels of tendon transcription factor genes SCX, Mohawk (MKX), Egr1, Six1, and Six2), were significantly reduced. Also The tendon-related genes Col Ⅰ and TNMD periostin and periostin were also downregulated (Wada et al., 2015).

### 4.2 Regulatory role of non-coding RNAs

#### 4.2.1 MicroRNA

To date, approximately 2,600 mature miRNAs have been identified in humans, and they play a role in regulating over 60% of coding genes (Liu Q. et al., 2021b). MiRNAs primarily function as silencers of coding genes by binding to the 3′-untranslated region (3′-UTR) of the target mRNAs,it either inhibits the translation process or facilitates the degradation of the mRNA. This mechanism regulates gene expression in a negative manner and is believed to be inversely related to the regenerative capacity of damaged tissues (Liu et al., 2019). MiRNAs play an important role in mediating tenogenic differentiation, apoptosis or senescence in tenocytes. MiRNAs regulate cytokine networks and control the proliferation and differentiation of stromal cells that affect the composition of the extracellular matrix.

Collagen formation is an important indicator of tenogenic differentiation occurring in TDSCs. MiR-378a inhibits tenogenic differentiatio by suppressing the production of Col I and ECM (Liu et al., 2019). MiR-29a has been identified as a potent post-transcriptional regulator with the capability to target Col I and III, making it a promising candidate for facilitating the repair of Achilles tendon injuries (Watts et al., 2017). MiR-29a also enhances the tendonogenic potential of TDSCs by regulating PTEN/AKT signalling and inhibiting TDSCs lipogenic differentiation and tendon fat infiltration (Yao et al., 2021).

The oxidative stress environment in AT leads to indiscriminate upregulation of anabolic and pro-apoptotic factors, and when the balance between the two is dysregulated, it causes increased miR28 expression and elevated levels of acetylated p53, leading to impaired antioxidant defences as well as activation of the pro-apoptotic pathway (Poulsen et al., 2014). In addition, miR-217 can also inhibit tenogenic differentiation of TDSCs by decreasing EGR1 expression (Han et al., 2017).

Elevated expression of Achilles tendon-specific markers in stem cells is closely related to their differentiation into tenocytes. MiR-135a promotes differentiation of TDSCs to tenocytes by upregulating gene expression with SCX and TNMD (Chen et al., 2015). In addition, miRNAs can regulate stem cell senescence. Notably, miR-335 has been linked to senescence in human MSCs, where it inhibits chondrogenic differentiation *in vivo* and markedly diminishes the *in vivo* immunomodulatory capabilities of hMSCs (Tomé et al., 2014).

#### 4.2.2 lncRNAs

In recent years, widely transcribed lncRNAs in mammalian genomes have been found to have multiple biological functions. lncRNAs regulate a wide range of biological events by modulating gene expression, including cell differentiation (Liu et al., 2023). Inflammation in Achilles tendon injury is closely related to collagen repair. The lncRNA FOXD2-AS1/miR-21-3p/PTEN axis is involved in the regulation of AT, and inhibition of FOXD2-AS1 reduces collagen fibre breakage, increases Col I and TNMD expression, and reduces IL-1β and TNF-α by modulating the miR-21-3p/PTEN axis and promoting the activation of the PI3K/AKT signalling pathway (Ke and Zhang, 2023). After co-culture of ADSCs and injured tenocytes, the expression of lncRNA Morf4l1 was upregulated and targeted to 3′UofmiR-145-5p to promote the expression of TGF-β2, inhibit oxidative stress and increase the cell proliferation capacity as well as to promote collagen repair (Zhao H. et al., 2022a). This process may be related to the demethylation of lncRNA.

Furthermore, it has been demonstrated that several lncRNAs, including H19 and KCNQ1OT1, can control TDSC differentiation. lncRNA H19 regulates tenogenic differentiation in TDSCs by directly targeting miR-29b-3p. Overexpression of H19 significantly upregulated the mRNA levels of tenogenic formation markers and ECM markers, including SCX, MKX, Col I, Fmod, TNMD, and DCN, and genetic silencing H19 led to a significant reduction in collagen formation and a decrease in the levels of these tenogenic formation and ECM markers (Liu Y.-J. et al., 2021c). Erroneous TDSCs differentiation is implicated in the pathogenesis of calcific tendinopathy, and knockdown of the lncRNA KCNQ1OT1 protects TDSCs from lipogenic and osteogenic differentiation by down-regulating the miR-138 target genes PPARγ and RUNX2 (Yu et al., 2018).

lncRNA MALAT1 regulates tenogenic differentiation of TDSCs through the miR-378a-3p/MAPK1 axis, and the expression of Scx, MKX, and Col I was downregulated after knockdown of lncRNA MALAT1 (Zhao Z. et al., 2022b). Some lncRNAs promote osteogenic differentiation of stem cells and thus act as promoters of AT. LncRNA AC108925, lncRNA MALAT1 and lncRNA TUG1, LncRNA HIF1A-AS2 may respectively promote osteogenic differentiation of TDSCs by regulating miR-146a-3p levels, RUNX2 expression, miR-665/IL6/PI3K/Akt pathway (Yu et al., 2020; Liu et al., 2023).

#### 4.2.3 circRNA

Circular RNAs (circRNAs) has a covalently closed loop and high stability compared to Col I, II, and III linear analogues, and have been identified as having a regulatory role in a variety of physiological functions (Han et al., 2022; Yang et al., 2023). CircRNAs regulate gene expression predominantly at the translational and post-transcriptional levels and can fine-tune cell proliferation, senescence and differentiation by interacting with RNA-binding proteins and/or miRNAs (Han et al., 2022). circ_0005736 enhances TGF-β1-induced TDSCs tenogenic differentiation by increasing SCX, MKX, and FMO as well as ECM (Col I) production through the miR-636/MAPK1 axis to promote TDSCs proliferation, invasion, and migration (Yang et al., 2023). circPVT1 plays an important anti-aging role in TDSCs. circPVT1 knockdown significantly inhibited the migration of TDSCs and reduced the expression of TNMD, Col I, and SCX, suggesting that circPVT1 is necessary for the regulation of migration and tenogenic differentiation of TDSCs (Han et al., 2022). Upregulation of circRNA-Ep400 expression by M2 macrophages during the inflammatory phase of Achilles tendon injury and upregulation of Col I, Col III and α-SMA as well as TGF-β1 expression via miR-15b-5p/FGF1/7/9 axes in fibroblasts and tenocytes promote accelerated peritendinous fibrosis after tendon injury (Jin et al., 2021).

## 5 Conclusion and future perspectives

The mechanical properties of the Achilles tendon render it particularly vulnerable to recurrent injuries and chronic dysfunction. In addition, the Achilles tendon tissue is rich in Achilles tendon matrix, lacks vascularity, and has a low cell count further adding to the challenges of healing tendinopathy. Currently, there are more exploratory applications of stem cells in rotator cuff and external epicondyle related diseases, while clinical applications carried out in the Achilles tendon are still in their infancy (Table 2). Inflammatory factor-induced mesenchymal stem cells or mesenchymal stem cell-based modulators of inflammation regulate inflammation levels and macrophage polarization after tendon injury (Wang S. et al., 2023). In the latest Phase IIa clinical trial, autologous bone marrow mesenchymal stem cell therapy relieved patients’ symptoms and improved walking ability (Goldberg et al., 2024). TDSCs are directly associated with M2 macrophage polarisation, both *in vitro* and *in vivo*, thereby attenuating the effect on pro-inflammatory signalling (Kouroupis et al., 2024). Obtaining both MSCs and TDSCs involves invasive manipulation of the donor, in addition to the scarcity of tendon tissue cells, which makes the process of culturing and expanding stem cells more difficult. Significant progress has recently been made in the study of iPSCs derived from somatic cells, providing a promising solution to the aforementioned challenges. Compared to TDSCs and MSCs, the application of iPSCs cells in the treatment of Achilles tendinopathy is still in its infancy, and the main focus is now on exploring the construction of more effective tendon differentiation protocols, the trajectory of iPSCs to tendon cell differentiation, and the changes in the expression of intermediary tendonogenic-associated cellular molecular markers. iPSC-tenocytes transplants can promote the motor function after Achilles tendon injury in rats through graft and paracrine effects recovery after Achilles tendon injury in rats (Tsutsumi et al., 2022). iPSCs have a theoretically unlimited proliferative capacity and large numbers of donor cells can be prepared relatively easily. In contrast, the collection of primary Achilles tendon stem cells or MSCs, especially from the elderly, often involves technical challenges. Second, cells derived from iPSCs tend to have juvenile characteristics. If function deteriorates due to aging, transplantation of juvenile cells would be a better therapeutic option (Nakajima et al., 2021). New materials for bioscaffolds are emerging, and scaffolds such as decellularised ECM hydrogels not only maintain the growth of TDSCs *in vitro* and protect TDSCs from shear forces during injection, but also provide a suitable microenvironment *in vivo* to promote the efficacy of stem cells. The development of suitable biomaterials to carry stem cells may be a better option than stem cell injection alone.

**TABLE 2:** Clinical trials of stem cells in tendons.

Cell type	Origin of cells	Type of tendon	Phases	References	Recruitment Status
MSCs	N/A	Rotator Cuff	Phase Ⅱ	NCT03362424	Suspended
	Bone Marrow	Rotator Cuff	Phase Ⅰ	NCT03068988	Closed
	Adipose-Derived	Lateral epicondyle	Phase Ⅰ	NCT01856140	Completed
	Adipose-Derived	Rotator Cuff	Phase Ⅰ	NCT06505135	Enrolling by invitation
	Adipose-Derived	Rotator Cuff	Phase Ⅱ	NCT01687777	Closed
	Adipose-Derived	Rotator Cuff	Pilot study	NCT02918136	Completed
	Adipose-Derived	Rotator Cuff	Pilot study	NCT04077190	Completed
	Adipose-Derived	Rotator Cuff	Phase Ⅱ	NCT02298023	Completed
	Adipose-Derived	Rotator Cuff, Lateral epicondyle	Phase Ⅱ	NCT03279796	Closed
	Adipose-Derived	Lateral epicondyle	Phase Ⅱ	NCT03449082	Closed
	N/A	Rotator Cuff	Pilot study	NCT02484950	Active, not recruiting
	Adipose-Derived	Rotator Cuff	Pilot study	NCT04670302	Closed
	Adipose-Derived	Lateral epicondyle	Phase Ⅰ, Phase Ⅱ	NCT02131077	Completed
	Adipose-Derived	Rotator Cuff	Pilot study	NCT03752827	Active, not recruiting
	Bone Marrow	Rotator Cuff	Pilot study	NCT06361797	Withdrawn
	Bone Marrow	Rotator Cuff	Pilot study	NCT03688308	Withdrawn
TDSCs	Achilles Tenden	Achilles Tenden	Phase Ⅱ	NCT02064062	Closed

Future studies could focus on the use of specific epigenetic regulators (e.g., DNMT inhibitors or histone deacetylase inhibitors) to target the promotion of tenogenic differentiation of stem cells, as well as standardisation of protocols for enrichment and maintenance of different tendon-derived cell populations. Although the current study initially explored the effects of non-coding RNAs (e.g., miRNAs, lncRNAs, circRNAs), their interactions in multiple epigenetic layers (e.g., binding DNA methylation and histone modifications) remain under-explored. Improved studies of the interactions of multiple epigenetic modifications may greatly assist in the treatment of Achilles tendinopathy and other diseases. Furthermore given that tendon ECM can regulate the fate of tendon stem cells, synergistic effects on stem cell differentiation can be studied by adding decellularized tendon ECM to the culture process.

The few clinical trials conducted so far did not perform *in vivo* follow-up after injection therapy, but rather evaluated by various rating scales and patient symptoms and activities. In order to advance the clinical application of stem cell therapy for Achilles tendinopathy, it is necessary to gain a deeper understanding of the mechanism of action and associated genetic epistemological modifications of stem cell therapy. Firstly, cells can be phenotyped and experimentally characterised using microscopy, qRT-PCR, flow cytometry sorting cell viability assay and immunofluorescence (Mechakra et al., 2022). The developmental trajectory of the implanted stem cells can be analysed using single-cell RNA sequencing, and genetic changes can be analysed by RNA-seq comparison and further validated by qPCR (Nakajima et al., 2021). Among them, sulfite sequencing is one of the most commonly used methods for DNA methylation analysis. And the development of high-throughput sequencing chromatin immunoprecipitation (ChIP-seq) has become the method of choice for analyzing histone post-translational modifications (PTM) and protein-genome binding. However, the methods currently used for epigenetic studies have some limitations. Bisulfite sequencing is highly sensitive and reproducible. However, bisulfite-treated DNA has only three different bases (A, T and G), and the lack of 285 cytosine bases reduces sequence complexity and hybridization specificity (Li and Tollefsbol, 2021). Chromatin immunoprecipitation with high-throughput sequencing (ChIP-seq) has been the method of choice for analyzing histone post-translational modifications (PTM) and protein-genome binding. The quality of sequencing data is largely dependent on the use of specific and efficient antibodies. Performing many parallel ChIP-seq experiments to compare different conditions, replicates, and epitopes is laborious and prone to experimental variation (Kumar et al., 2024).

The activities of stem cells such as differentiation, self-renewal, and immunomodulation are regulated by multiple factors at the target site. When stem cells were transplanted into the site of Achilles tendon injury, stem cell viability may be compromised due to inflammatory infiltration of the microenvironment, ECM disturbances, and insufficient blood supply for nutrients, as well as impairing the repair of AT due to differentiation into other cell types such as osteoblasts and lipocytes and interfering with Achilles tendon healing and biomechanical properties by generating faulty ECMs *in vivo*, as well as the presence of the possibility of cellular tumorigenicity. Previous work has acknowledged the ability of MSC and iPSCs to form teratomas at the injection site, and future work should consider the risk of insertional mutagenesis and alterations associated with initiation to limit the generation of tumorigenesis (Huerta et al., 2023; Lin et al., 2024). The tumorigenic potential of iPSCs can be decreased by optimizing the cocktail of reprogramming factors using the following strategies: (1) chemical reagentinduced reprogramming, (2) purification of iPSCs, (3) exogenous DNA-free vectors, and (4) nanoparticle and thermos-responsive polymers (Lin et al., 2024). Further clarification of the biological niche of the stem cell niche and injection of the stem cells into the appropriate site can help in the tendonogenic differentiation of the stem cells.

Stem cell therapy for AT needs to be approved and regulated by the FDA (U. S. Food and Drug Administration), EMA (European Medicines Agency), UK Medicines and Healthcare Products Regulatory Agency (MHRA) and PMDA (Pharmaceuticals and Medical Devices Agency of Japan). Harmonizing local regulations and related technologies to shape safer and more effective stem cell therapies is critical to expedite the approval of clinical trial access. The extraction, expansion, transportation, and injection of stem cells need to follow strict standards to ensure safety, purity, and consistency. Cells from the same batch need to maintain uniform genetic modifications. Additionally, the application of genetically modified modulators requires extensive preclinical studies to demonstrate efficacy and long-term safety.
